# XFEL Beamline Optical Instrumentation for Ultrafast Science

**DOI:** 10.1021/acs.jpcb.4c01492

**Published:** 2024-08-01

**Authors:** Christopher D. M. Hutchison, Samuel Perrett, Jasper J. van Thor

**Affiliations:** † Department of Life Sciences, Faculty of Natural Sciences, Imperial College London, London SW7 2AZ, United Kingdom

## Abstract

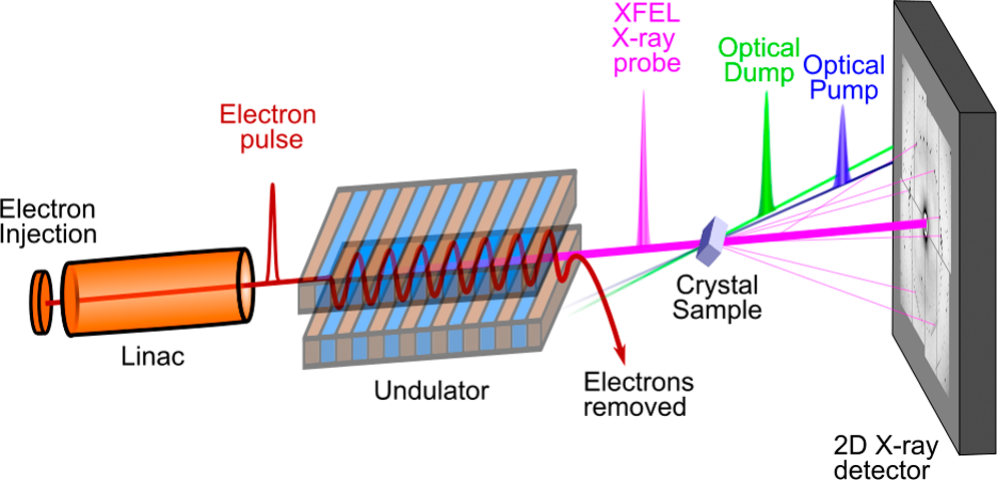

Free electron lasers operating in the soft and hard X-ray regime provide capabilities for ultrafast science in many areas, including X-ray spectroscopy, diffractive imaging, solution and material scattering, and X-ray crystallography. Ultrafast time-resolved applications in the picosecond, femtosecond, and attosecond regimes are often possible using single-shot experimental configurations. Aside from X-ray pump and X-ray probe measurements, all other types of ultrafast experiments require the synchronized operation of pulsed laser excitation for resonant or nonresonant pumping. This Perspective focuses on the opportunities for the optical control of structural dynamics by applying techniques from nonlinear spectroscopy to ultrafast X-ray experiments. This typically requires the synthesis of two or more optical pulses with full control of pulse and interpulse parameters. To this end, full characterization of the femtosecond optical pulses is also highly desirable. It has recently been shown that two-color and two-pulse femtosecond excitation of fluorescent protein crystals allowed a Tannor-Rice coherent control experiment, performed under characterized conditions. Pulse shaping and the ability to synthesize multicolor and multipulse conditions are highly desirable and would enable XFEL facilities to offer capabilities for structural dynamics. This Perspective will give a summary of examples of the types of experiments that could be achieved, and it will additionally summarize the laser, pulse shaping, and characterization that would be recommended as standard equipment for time-resolved XFEL beamlines, with an emphasis on ultrafast time-resolved serial femtosecond crystallography.

## 1 Introduction

X-ray free electron lasers (XFELs) provide many types of time-resolved experiments. Instruments that operate only in the soft X-ray regime currently include FLASH (Hamburg, Germany),^1,2^ FERMI (Trieste, Italy),^3,4^ and SXFEL (Shanghai, China).^5^ Currently operating XFEL facilities in the hard X-ray regime are LCLS (Stanford, USA), SACLA (Japan), PAL-XFEL (Korea), SwissFEL (Switzerland), and Eu-XFEL (Hamburg, Germany), all of which have additional soft X-ray beamlines. LCLS-II currently provides soft to tender X-rays, but the future LCLS-II-HE upgrade will be a hard X-ray source. A wide range of femtosecond time-resolved techniques at these facilities have helped to probe the fundamental light-induced processes in both chemical and biological systems. Briefly, we will summarize the main techniques which can broadly be split into two domains; spectroscopic and scattering.

X-ray absorption spectroscopy (XAS) at FELs allows element specificity with binding energies that are sensitive to the local chemical environment. Sharp absorption edges are observed when scanning X-ray photon energy in the vicinity of a particular atom core electron binding energy. XAS has successfully tracked light-induced dynamics on the sub-100-fs time scale, such as the first ionization events of liquid water.^6^ X-ray absorption near-edge structure spectroscopy (XANES) is particularly effective for analyzing the electronic and geometric structures of samples and uniquely capable of probing d–d electronic transitions in excited states that are not detectable by optical spectroscopy. Furthermore, it can probe metal-to-ligand charge transfer (MLCT) and spin crossover dynamics^7,8^ and nuclear dynamics^9^ in metal complexes. This technique, applicable in both condensed and gaseous phases, effectively reveals details about valence states and low continuum states near the ionization threshold by measuring changes in oxidation states, molecular symmetries, and lowest unoccupied molecular orbitals (LUMO)s through photoabsorption cross sections at various X-ray energies. EXAFS (extended X-ray absorption fine structure) measures beyond the absorption edge and differs from XANES by measuring modulations of the absorption coefficient resulting from the photoelectron emitted during the ionization event. This liberated photoelectron scatters off neighboring atoms, causing interference in the spectra at greater energies than the edge.^10^ EXAFS allows the precise measurement of structural details such as bond lengths and coordination numbers around a central atom, offering insights into atomic arrangements.^11−13^ Unoccupied states can be probed in a single-shot manner by high energy resolution off-resonant spectroscopy (HEROS).^14−16^ This technique enables the capture of a scattered X-ray spectrum in one acquisition using a monochromatic incident beam.

X-ray emission spectroscopy (XES) differs from XAS in measuring emitted photons from fluorescence following ionization. The most common XES measurement is from core 1s ionization, for which numerous peaks in the XES spectra will be observed from various decay pathways 2p → 1s (Kα), 3p → 1s (Kβ), etc. Splitting of the peaks due to spin–orbit coupling and electron exchange can occur, and such contain information on the oxidation and the spin state.^17^ This makes XES particularly useful in transition-metal complexes where the spin sensitivity of the technique can capture intermediate spin states and spin crossover dynamics.^18−20^ The complementary techniques of XAS and XES can routinely be performed in situ at FELs.^21^

Small-angle scattering and wide-angle scattering (SAXS/WAXS) are used to garner information on the shape and size of molecules, from small molecules^22^ to macromolecules on the order of tens to hundreds of angstroms. Differing only in their measurement angle, they offer structural information on the nanoscale and have been used to observe structural rearrangements such as protein quakes and can be used to measure helical motions, hydrophobic collapse, and allosteric regulation.^23−26^

Diffractive techniques in the pump–probe geometry allow for direct recording of structural changes in materials and have been highly successful at XFELs. Time-resolved serial femtosecond crystallography (TR-SFX) has captured numerous structural molecular movies of biologically relevant targets^27^ including photoactive yellow protein (PYP),^28−30^ photoswitching derivatives of green fluorescent protein,^31−33^ rhodopsin,^34,35^ photosystem II,^36,37^ ribonucleotide reductase,^38^ myoglobin,^24^ and photolyase.^39,40^ TR-SFX has the caveat of requiring crystalline samples. At FELs, it is performed in a serial manner, where a new crystal is introduced for each new X-ray shot. A diffraction pattern is recorded before the crystal is destroyed, predominantly due to ionization events.^41^ By combining tens of thousands of frames, one can reconstruct a 3-D structural model. By combining this with a “pump” to initiate a reaction and delaying the hard X-ray probe, a series of structures can be solved along the reaction coordinate. An example is a recent high-resolution (1.35 Å) ultrafast optical control TR-SFX experiment, which assigned contributions from coherent motion.^42^ Recently, TR-SFX has moved to the chemical domain.^43−45^

Resonant inelastic X-ray scattering (RIXS) is a photon-in, photon-out scattering process in which a photon excites a core electron to an intermediate state, which then relaxes to a lower energy level, emitting a photon. RIXS has revealed dynamics in ligand exchange,^46^ localized electronic transitions,^47^ and magnons.^48^

Over the past decade, XFEL science has matured, culminating in the array of pump–probe techniques mentioned that probe the broad spectrum of dynamics in molecular, biological, and material fields. All of these techniques utilize a short laser pulse to trigger the desired dynamics, making it an essential part of the experimental design.

One of the earliest XFEL beamlines that was commissioned to perform pump–probe hard X-ray experiments is the X-ray pump–probe (XPP) beamline at LCLS in 2009 and 2010. The beamline utilized a core laser configuration that remained essentially unchanged at the time of writing. This setup includes a 120 Hz titanium:sapphire laser and a TOPAS optical parametric amplifier (OPA), capable of delivering UV, visible, and near-IR wavelengths with pulse durations typically around 50 fs and pulse energies ranging from microjoules to millijoules, matching the LCLS pulse repetition rate. However, despite the core laser capability remaining constant, there have been numerous additions and enhancements over the years. These improvements encompass various multipulse excitation schemes, THz generation using organic crystals, UV extension down to 200 nm, the generation of few-cycle pulses via hollow-fiber pulse compression, enhanced temporal resolution through time-tool developments, and the integration of nanosecond systems.^49^ Ultimately, the specifications of laser systems for XFEL beamlines will need to match the repetition rate of the X-ray pulse train, the detector rate, and the requirements for the optical parameters that must be considered on a single shot basis.

The superconducting XFEL facilities Eu-XFEL and LCLS-II (including the future hard X-ray LCLS-II-HE) are MHz repetition rate machines. The macrobunch structure of the Eu-XFEL perhaps presents the most challenging conditions and therefore requirements of an optical laser source to support ultrafast studies. The development and operation of the high-end burst laser system at Eu-XFEL^50−52^ is a good example of current technology and limits to application.^53,54^ The PP laser system is capable of running up to a 4.5 MHz repetition rate, and the fundamental was designed to be at 800 nm, supporting pulse durations of as short as 15 fs in the fundamental. Frequency conversion, besides SHG and THG, is done using conventional colinear OPAs that can support up to a 1.1 MHz repetition rate at Eu-XFEL. This system is, however, designed to operate in burst mode in order to match the Eu-XFEL pulse profile itself. For continuous MHz repetition rate operation, at sufficient pulse power and pulse duration, it will be necessary to employ different laser technologies to achieve these goals.

The LCLS-II synchronized optical laser system will use optical parametric chirped pulse amplification (OPCPA) technology, capable of supporting 35 W, 800 nm sub-20 fs pulses with 0.4 mJ at the 93 kHz nominal repetition rate of LCLS-II or 1 mJ at 33 kHz. This will service the first two soft X-ray end stations (TMO-IP1 and chemRIXS) when they come online this year. Using Ytterbium (Yb) technology for the pump lasers at 1030 nm, a series of OPCPA stages (manufactured by R&D systems) provide high output of >150 W in the near infrared (NIR) and 100–200 W in the infrared (IR) spectral region. This power level is likely typical and close to maximum performance of the type of source that should be considered for further frequency conversion, shaping, and characterization methods that we propose here. Even more powerful OPCPA configurations exist, such as the systems installed at ELI-Beams, Prague, where the L1-ALLEGA laser delivers 30 mJ, 15 fs pulses but is limited to 1 kHz^55,56^ while the other L2, L3, and L4 systems^57^ deliver, or plan to deliver, even higher pulse energies but in each case at the cost of the repetition rate.

For the remaining “near experimental hall” end stations of LCLS-II, there is the plan to use spectral broadening and compression of the 1030 nm pump laser of the OPCPA systems. This aims to provide <30 fs, 1.5 mJ at 33 kHz which can be combined with a variety of wavelength conversion schemes including harmonic generation, OPA’s, and DFG with possible additional pulse compression attached to each.^49^ Pulse energies of around 1 mJ are sufficient for most applications, although frequency conversion may present an additional limitation for high repetition rate experiments due to thermal limitations. However, for a specialized subset, mainly high-energy far infrared (FIR) and THz generation, pulse energies of approximately 10 mJ would be preferable. Efforts to increase the power output to several hundred watts are currently under active research and development.^49^ Other possible plans include optical rectification, resonant dispersive wave (RDW), and four-wave mixing. To make best use of the ultrashort pulses, further efforts to synchronize the optical laser systems with the XFEL including radio frequency (RF) locking, optical locking using a pulse fiber timing system from cycle lasers (similar to that currently deployed at SACLA^58^), and multiple arrival time monitors.^59^ A similar approach is planned for the hard X-ray stations in the “far experimental hall” as part of the developments for LCLS-II-HE.^49^

The experimental configurations for ultrafast pump–probe-type studies should be considered at the level where XFEL facilities are able to provide the basic specifications and configuration for the laser fundamental, which will determine the frequency conversion methods and performance. In practice, the examples of the Eu-XFEL burst laser and the LCLS-II OPCPA source do provide sufficient optical power levels for typical pump–probe studies which have been demonstrated for the lower repetition rate machines (LCLS, SACLA, SwissFEL, and PAL-XFEL). At these facilities, titanium:sapphire laser technology with conventional OPAs is typical.

For experiments that need extensive pulse shaping and the generation of pulse replicas or pulse trains and frequency conversion, the selection of the carrier frequency of the fundamental laser system should be carefully made. It is, of course, not possible to specify a single laser system that could satisfy all possible user requirements. However, the OPCPA technology is particularly attractive, which has the ability to provide high power, high repetition rates, and short pulse operation. The necessary frequency conversion and shaping processes will inevitably reduce the available optical power density on the sample and always will exhibit a wavelength dependence in efficiency.

In addition to the XFEL and pump laser repetition rate, the detector frame rate places another practical limit on the maximum data rate an experiment can achieve. Diffraction studies, in particular ultrafast crystallography, require large (>1M) 2D area detectors with high dynamic range. While semiconductor technology does support MHz frame rate capturing of megapixel area detectors, the data throughput is the true bottleneck. Such high repetition rates are typically possible only in burst mode. This involves the use of electronic buffers which are filled at the maximum repetition rate and then must be periodically emptied to transfer the data out. Technologies that allow MHz continuous frame rates exist, but it may not be possible to record each data frame at the level of TB/s generated from megapixel area detectors. One possibility is to use field programmable gate array (FPGA) devices to preprocess and reduce data streams. Another approach which has been developed for LCLS-II is to allow a MHz continuous frame rate and GHz burst rate performance using an application specific integrated circuit (ASIC) to reduce the data. The SparkPix-ED or SparkPix-S is an integrated ASIC that can select rare events and reduce the data rate from TB/s to GB/s for specific applications.^60^ The system uses a low-resolution pixel-sum approach to detect rare events and enables a high-resolution readout.

The ePix X-ray area detectors developed at SLAC National Accelerator Laboratory have various modes that can support beyond a 1 kHz continuous frame rate.^61,62^ The Jungfrau hybrid integrating area detector developed by the PSI supports up to a 2 kHz rate. At the Eu-XFEL, the AGIPD^63,64^ and LPD^65,66^ have been developed to support up to 4.5 MHz in burst mode. The commercial Rigaku XSPA-500 K^67^ supports a 56 kHz continuous frame rate and a 1 MHz frame rate in burst mode. The XSPA-500 K is, however, a hybrid photon counting detector, which has a paralysis time of hundreds of nanoseconds and is not suitable for measuring femtosecond signals at XFELs. Detector systems will need to be developed that support the full MHz repetition rate of XFEL machines, and these must be based on integrating technology and deliver a high dynamic range. At Stanford Linear Accelerator Center (SLAC), the long-term area detector program has created a series of detector designs in the ePix family to allow kHz to GHz image capture. This means that laser technology could in time become the limiting factor of the overall data collection rate. Assuming that a “comfortable” pulse power level of 1 mJ is taken as a basic requirement for the fundamental of the system, LCLS-II would need a 1 kW OPCPA to run continuously. We will assume this power level of 1 mJ for single pulses for the proposed beamline instrumentation, needed to ensure frequency conversion and shaping schemes for experimental conditions, which are discussed below. This Perspective is structured as follows: 1) We will briefly review a recent and first demonstration of coherent control of vibrational dynamics shown by X-ray crystallography. 2) We will outline, in overview rather than exhaustive detail, the types of physical measurements that could be enabled by adding pulse-shaping capabilities to beamline instrumentation. 3) We will briefly summarize the optics instrumentation to allow both the shaping and the diagnostics that would be required for such studies.

## 2 Demonstration of Optical Control of Vibrational Coherence in a Light-Sensitive Protein

Since the first demonstrations of femtosecond time-resolved pump–probe protein X-ray crystallography on Myoglobin in 2015^68^ and the PYP in 2016,^30^ quite a number of additional examples have been shown.^31,35,69,70^ Until recently, most of these studies have been interpreted essentially using rate kinetics arguments. It is an open question of under which conditions such methods are applicable on the ultrafast time scale. In reactive systems that include a conical intersection, such as the examples of photoisomerization in biological and chemical systems, the key consideration is whether femtosecond time-resolved snapshots would measure the motion on the S_1_ surface, the passage through the conical intersection, and/or the product state formation. First, the photoisomerization would need to be vibrationally coherent for this to be expected. This has been proposed for the photoreactions of rhodopsin in human vision.^71,72^ In rhodopsin, this was proposed from the observation of vibrational coherence in the ground-state photoproduct on short time scales, and this idea is still being debated in detail. From the perspective of theory, a very detailed modeling of the quantum dynamics must support such an assignment, including the applications of Engleman-Jortner theory to understand the branching ratio in the conical intersection. Furthermore, it should be considered that Landau–Zener surface crossing itself creates coherence,^73,74^ a property that is also exploited in the TRUECARS technique developed by Mukamel et al.^75^ Generally, developing the evidence for vibrationally coherent photoisomerization is a lengthy and challenging process, but it should be established prior to the analysis of X-ray crystallographic difference density dynamics. Most examples involving biological photoisomerization involve an incoherent thermally activated barrier crossing in the excited state, and as such the kinetics would show a temperature dependence in the incoherent case. This should, however, be shown over a very large temperature range since any deviation of true Arrhenius kinetics would alter the appearance of the activation energy in a smaller region. Examples for photoswitching fluorescent proteins have recently been shown.^42,76^ Direct evidence for incoherent excited state barrier crossing was recently reported for synthetic photoswitching fluorescent protein “rsKiiro”.^42^ In this example, it was found that at ambient temperature the photoisomerization appears as an almost barrier-free rotor motion, while a sizable barrier in fact exists and is revealed from the non-Arrhenius kinetics at low temperature. Incoherent thermally activated barrier crossing was furthermore evidenced from femtosecond action spectroscopy of the crystals. The measurements combined femtosecond pump and dump pulses, resonant with the ground-state absorption and stimulated emission, respectively. From scanning the pump–dump delay times, the cross correlation of the pulse envelopes was found; furthermore, it was seen that the depletion of photoisomerization, due to the dump pulse, followed the 50 ps excited-state decay. Therefore, the ground-state photoproduct accumulates statistically over the period of the excited state decay, and the actual motion involved in the reaction coordinate is not observed in the ensemble measurement. In the incoherent case, the time-dependent measurements show differences in the concentration of the product state only, and conventional rate kinetics can be used.

Protein X-ray crystallography is blind to electronic excitation, and the evidence for coherent or incoherent dynamics is additionally not straightforward to obtain experimentally. It is therefore very helpful to include additional time-resolved measurements that include a stimulated emission pumping (or “dumping”) interaction directly after a “pump” interaction. It was shown that for stimulated emission pumping at power density comparable to that of the pump, the S_1_ state in crystals of the rsKiiro fluorescent protein could be fully depleted, which subsequently switched off the photoisomerization reaction.^42^

Performing the pump–dump–probe in addition to the conventional pump–probe time-resolved X-ray crystallography measurements led to the discovery that ground-state vibrational coherence dominates the early (<1 ps) time electron density differences in the rsKiiro crystals.^42^ The relatively large displacements that were seen in the chromophore region and throughout the protein core indicated coherent ground-state motions across both wells in an adiabatic double-well potential. The contributions of excited-state motions were in fact not resolved. These are not generally valid conclusions, since difference electron density signals depend on many parameters including the excited-state displacements, ordering parameters including heterogeneity and Debye–Waller factor, the scattering cross sections, and the magnitude of the resulting real-space displacements. Nevertheless, it was previously argued on the basis of theoretical considerations that the ground-state vibrational coherence is expected to be very large under typical conditions of a TR-SFX experiment. This is because it is population-driven and the experimental conditions often include, because of the signal-to-noise ratio, intense optical pump power.^77,78^

The assignments of vibrational coherence and its transfer during the stimulated emission pumping were directly taken from the literature on impulsive stimulated Raman spectroscopy (ISRS) as well as the theoretical considerations for Tannor-Rice coherent control.^77,79−81^ A simple four-level system was used as a model to discuss and demonstrate the quantum dynamics of the rsKiiro fluorescent protein. It contains two vibrational states in both a ground and excited electronic state, with the vibrational frequency set to 170 cm^–1^ for both levels. The excited-state levels were set such that the 400 nm experimental pump could populate both vibrational levels of the excited state (|2⟩ and |3⟩) from, predominately, the vibrationally cold ground electronic state (|0⟩)

**Figure 1:**
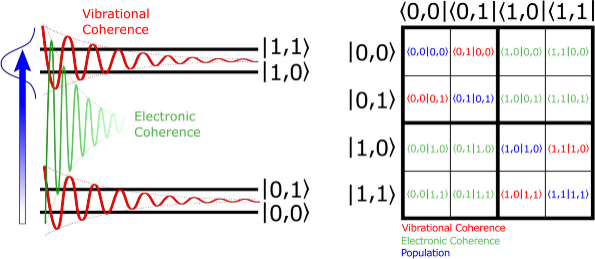
A basic and simplified model for coherence and population dynamics following the optical excitation of HOMO/LUMO transitions, including vibrational excitations (left). An applicable density matrix with elements separating the coherences and populations is shown (right). This Perspective focuses on optical control and manipulation of these dynamics in combination with ultrafast XFEL probes.

The first pump interaction prepares population transfer from the vibrational ground state of S_0_ into the two lowest vibrational states of S_1_ (i.e., ρ_00_ → ρ_22_ + ρ_33_) (Figure 1) and creates ground-state coherence between the vibrational states in S_0_ (i.e, ρ_01_ from interaction with the Boltzmann distributions of ρ_00_ and ρ_11_ as well as excited-state coherence ρ_23_ using the experimental values for the laser spectrum and pulse duration). We introduced the Stokes field with a 350 fs pump–dump delay according to the experimental parameters and using the criterion Γ_electronic_ > 1/Δ*t*_pump–dump_ > Γ_vibrational_. The carrier frequency couples the population transfers and efficiently transfers the vibrational coherence ρ_23_ → ρ_01_. Simulations of this process were carried out using a nonperturbative density matrix calculation and were subsequently analyzed in phase space by performing Wigner phase space transforms to the density matrix.^42^

The summary given here is the first demonstration of a multipulse optical excitation experiment with results analyzed from X-ray crystallography in real space. The XFEL experiments required specific pulse parameters based on home laboratory spectroscopic measurements, and whatever femtosecond laser was resident at the beamline was set up to approximate home laboratory conditions as closely as possible. Furthermore, full laser pulse characterization, which is not generally available at any XFEL facility to the best of our knowledge, was done for our experiments using user-provided equipment. Pulse-shaping capabilities and characterization are not typically available at XFEL beamline instruments at the time of writing. We were able to create a two-color experiment by synthesizing a 400 nm pulse by second harmonic generation (SHG) of the titanium:sapphire lasers at the LCLS/CXI and SACLA/EH2 beamlines and dump pulses at 515 nm with the available OPAs. Therefore, we were able to perform a pump–dump–probe three-pulse (two optical, one X-ray) experiment using beamlines that provide only the basic equipment for a pump–probe experiment. Under such circumstances, shaping is realistically limited to passive stretching, verified by X-FROG, which in the case of the TR-SFX experiments of PYP crystals was necessary to suppress nonlinear excitation and maximize the photoisomerization yield.^30^ The optical setup that was done takes time away from data collection and is usually done during limited time slots given to users during off shifts or prior to or even after the official allocated beamtime.

In our view, XFEL beamlines that support ultrafast studies should provide as standard 1) the capabilities for compression and/or shaping of the pulse duration, spectral and temporal phases, and bandwidth; 2) the capability to synthesize multipulse single-color and/or two-color pulse trains and combinations and cross-correlation and timing of the final pulse train; and 3) characterization, at a minimum, of pulse duration from some correlation technique but ideally full characterization using FROG, or equivalent, techniques for all possible outputs. 4) In addition we propose an easy add-on capability to allow a rapid Z-scan characterization which for many users will confirm the optimization of nonlinear cross sections in a matter of minutes, with minimal additional instrumentation (see below). At the time of writing, LCLS is actively pursuing the implementation of technologies such as pulse stacking, shaping, and characterization.^49^

## 3 Connection among Nonlinear Optical Spectroscopy Techniques, Nonlinear X-ray Science, and Ultrafast XFEL Techniques

In this Perspective, we focus on the application of ultrafast nonlinear optical and vibrational spectroscopy techniques to include X-ray experiments for detection. As outlined in the example above, ultrafast laser pulses prepare electronic coherences, involving the valence electrons, and nuclear coherences (Figure 1). The resulting nonlinear response involves the Raman selection rules. Of course, in the field of nonlinear spectroscopy the molecular response is written as the resulting optical polarization. For X-ray experiments such as XAS, possible applications that combine phase-locked optical laser pulses should retrieve a higher-order response that involves the coherence path for both core and valence electrons. This is one of the possible very interesting applications of mixed four-wave-mixing experiments proposed here. For this analysis, all electronic levels must be put in the same manifold, which is in principle the case. Related experiments include the development of hard X-ray transient grating experiments that have been shown at XFELs^82,83^ and include the combination of X-ray and optical fields. Recently, X-ray pump–probe techniques in the XUV region have been demonstrated that measure electronic coherence via wavepacket interferometry and strong field quantum control. These results demonstrate the absorption spectroscopy principle of nonlinear response that involves core electron excitation.^84−86^ An extension of these techniques to higher-order responses can be envisioned that includes a multiple combination of X-ray pulses only^84^ or indeed mixed optical and X-ray applications. X-ray scattering and crystallography experiments contain real-space information for which the higher-order response cannot directly be written using response function formalisms. The effective selection rules for detection are unconnected to the polarization of the emitted field in the formal sense of analysis if scattering or crystal diffraction is involved. Both approaches have the ability to directly measure vibrational coherences^42^ and, in selected cases, valence electron dynamics.^22^ In order to make an analysis of coherence dynamics, we have previously shown that Wigner transforms of time-dependent density matrix calculations provide a more direct methodology because the transformed complex amplitude is expressed in phase space and can thus be directly compared to the experimental phase space observations.^42,87^ In principle, there are a multitude of pulse sequences involving both optical and X-ray pulses that can probe the nonlinear molecular response. A nonlinear optical response that results in high harmonic generation (HHG) is yet another field and also is not explicitly considered here.^88^ In this Perspective, we focus on opportunities that exploit the optical laser excitations to prepare and control coherences as shown in Figure 1.

## 4 Birds-Eye View of Possible Multipulse Applications for Ultrafast XFEL Science and Structural Dynamics

The following summarizes in brief overview the possible pulse schemes that can be proposed and the structural dynamics that can be interrogated. The dedicated optics instrumentation to be installed are discussed in section 5. The typical configuration of current XFEL beamlines includes a single-color femtosecond optical excitation for pump–probe studies. Under conditions of electronic resonance, the pump generates population and coherences, specifically electronic and vibrational coherences including impulsively created ground-state coherence (Figure 1). With this application, it is very challenging to separate the ground-state and excited-state vibrational coherence contributions. As is done in the field of impulsive stimulated Raman spectroscopy (ISRS), with an accurate determination of the coincidence time, a phase analysis of periodic displacements could provide a level of information that can aid the assignment of individual modes. Furthermore, the carrier frequency and to some extent also the phase may inform assignments.^73,74,89−92^ A direct extension of the ISRS technique applies optical control via chirping of the femtosecond pulse in order to control the amplitude of the nuclear coherence. Bardeen et al. showed examples where negatively chirped pulses were shown to enhance the ground-state coherence whereas positively chirped pulses suppressed the amplitude.^93^

**Figure 2:**
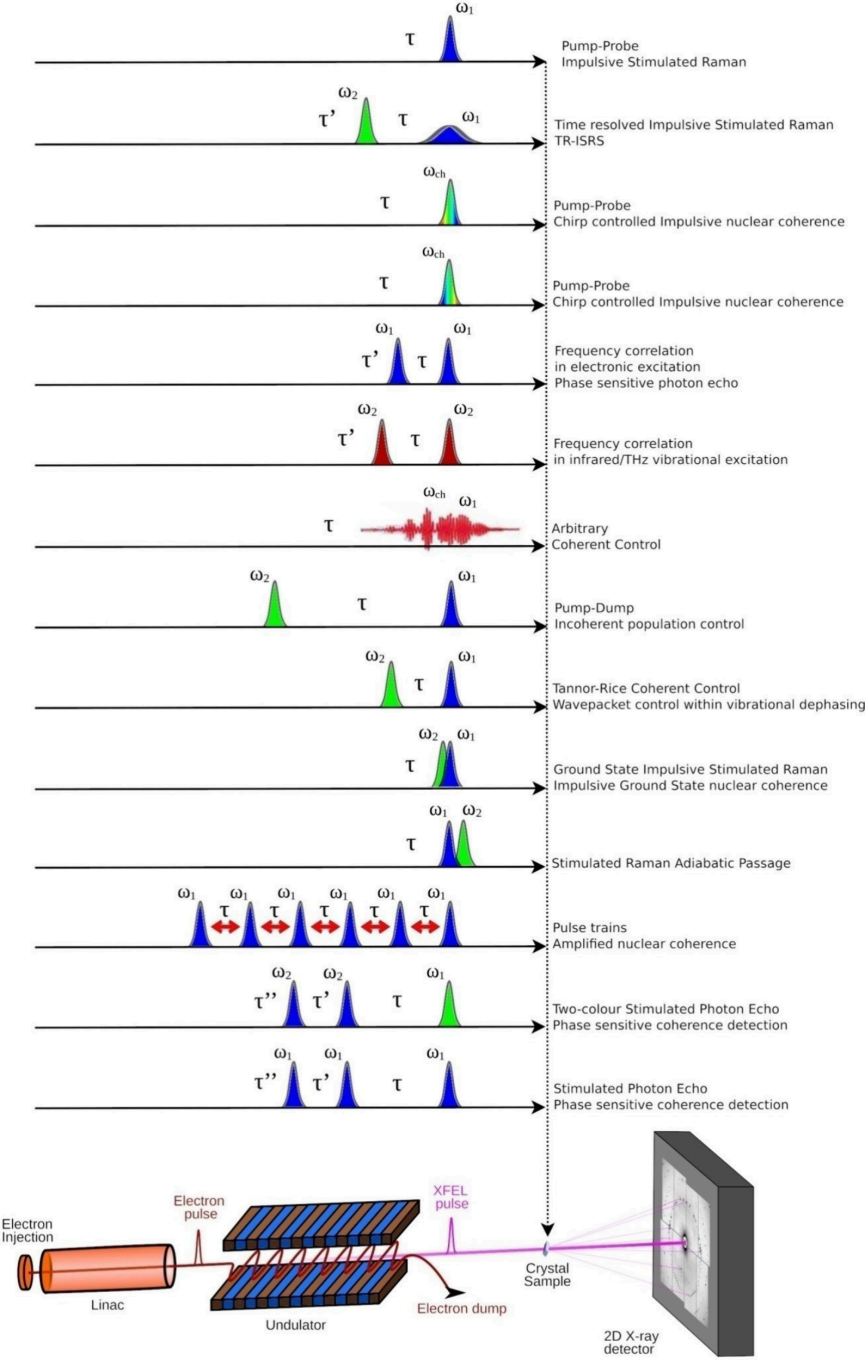
Overview of proposed pulse schemes for ultrafast hard X-ray diffraction, taken from nonlinear optical spectroscopy and coherent control methods.

Another direct modification of the ISRS methodology uses a two-pulse and two-color scheme (Figure 2), where an initial “actinic” pulse is a narrow-bandwidth and stretched pulse, typically a few picoseconds duration and below the 10 cm^–1^ spectral width, which is combined with a short Raman pulse which is in resonance with excited-state absorption (ESA) or the stimulated emission (SE)-induced cross section. The “TR-ISRS” methodology relies on the initial preparation of the population which is frequency-limited for the ground- and excited-state vibrational coherence. Therefore, all high-frequency coherence that is observed in the experiment following the Raman pulse is specific for transient excited state processes.^94^ Spectroscopy investigations have addressed the precise details of the optical and molecular parameters to describe the contributions to the conventional (pump–probe-type) ISRS response, which is beyond the scope of this discussion.^95−97^ The literature on femtosecond Raman spectroscopy is very broad and complex, with different experimental approaches to time domain and frequency domain measurements. Of course, this literature is relevant to the spectroscopic measurements of the Raman effect and at minimum follows the four-wave mixing response of the full coherence treatment but has been extended to include a higher-order response. Here, we borrow components from the literature in order to propose the equivalent ultrafast X-ray crystallographic measurements of the nuclear coherences that are prepared with femtosecond pulse schemes. It should be emphasized that the Raman selection rule does not apply to the observation that is considered here. Instead, the detection of crystallographic differences depends on the crystallographic ordering and displacement parameters and X-ray scattering cross sections. Nevertheless, the creation of electronic and nuclear coherence is well described in the Raman spectroscopy literature and can be directly applied to ultrafast X-ray crystallography.

Two-dimensional spectroscopy provides more information than pump–probe spectroscopy, principally by providing the frequency correlation of the excitation. A typical experimental configuration applies two phase-locked pulses where a variation of the interpulse delay creates the frequency axis for the spectroscopic response.^98^ In 2D spectroscopy, a third pulse is applied, and the emitted four-wave mixing signal is either homodyne or heterodyne detected with frequency dispersion. Here, we propose a time-resolved X-ray experiment where instead of a single pump pulse a phase-locked pulse pair is used. By varying the interpulse delay in a phase-stable manner, a frequency correlation of the time-resolved crystallographic differences can be created. In this proposed experiment, two delays will need to be incremented: both the phase-stable interpulse delay and the waiting time. This will create a five-dimensional data set where the three-dimensional electron density differences can be analyzed as a function of either delay. The waiting time dependence would be equivalent to the conventional time-resolved crystallographic “molecular movies” reported in the literature already,^30,31,42,69,70^ whereas the scanning of the interpulse delay would reveal the frequency correlation of the electron density signals for each waiting time (Figure 2). In the conventional four-wave mixing formalism, the first pulse creates an off-diagonal matrix element or coherence, and the second pulse switches the density matrix into a population state after which population decay proceeds and could be probed with time-resolved X-ray diffraction. By adding a third pulse with another delay, the density matrix is switched back into a coherent state, after which the emitted third-order response that gives rise to the signal field of the third-order optical polarization is measured using spectroscopic detection. Figure 2 includes possible examples for photon echo-type experiments, where the phase matching condition is  and the stimulated photon echo which involves an additional interaction with phase matching condition . Variations could include the reverse photon echo, where an inversion of the time ordering is used, with phase matching condition . In the photon echo, the last two interactions occur simultaneously; in the reverse photon echo, the two simultaneous interactions occur first. An equivalent X-ray diffraction experiment could map the structural dynamics that correspond to the full four-wave mixing pulse sequence with the three-pulse equivalent of the stimulated photon echo measurement (Figure 2). Experiments that include XAS should follow the phase matching conditions, whereas scattering and diffraction experiments are not selective for the emitted field. In principle, the same type of information can be obtained by using resonant infrared or THz excitation of molecular vibrations, where the frequency correlation of the molecular modes would be retrieved instead (Figure 2).

The coherent control literature offers multiple approaches in order to propose novel pulse schemes for time-resolved XFEL diffraction (Figure 2). As with ISRS, a complete discussion of this literature is beyond the scope of this text. The method of coherent control is well established in the literature. An example can be highlighted, where computational approaches have been used with the aim of achieving coherent control of the photoisomerization dynamics of rhodopsin by using pulse shaping.^99^ The analysis is very attractive as it deals with a wavepacket calculation for passage through a conical intersection to control the nonadiabatic dynamics. The mechanism of optical control was described as a “wave packet cannon” by Abe and Domcke,^99^ who additionally describe directly related literature in their work. Experimentally, it may be proposed to apply optical control techniques via active pulse shaping, where complex pulses could be optimized using spectroscopy techniques and subsequently directly applied at XFEL beamlines using computer-controlled shaping devices such as the acousto optic programmable dispersive filter (OAPDF, Dazzler^100^) (Figure 2). A Tannor-Rice pump–dump control was recently demonstrated with TR-SFX.^42^ This type of scheme uses a two-pulse, two-color experiment where the Stokes pulse arrives within the vibrational dephasing time. This type of control has been shown to transfer vibrational coherence from the excited state to the ground state, which resulted in a strong amplification of coherent nuclear motion in the ground state on picosecond time scales.^42^ By shortening the delay between the pump and the dump pulses to within electronic dephasing, typically less than 50 fs in biomolecules, the stimulated Raman process would be selected (Figure 2). Effectively, this would be equivalent to the case where negatively chirped single pump experiments (at high intensity) drive stimulated Raman to create ground-state nonstationary motion, as originally shown by Bardeen et al.^93^

By moving the Stokes pulse so that it arrives before the pump pulse, the process of stimulated Raman adiabatic passage (STIRAP)^300,301^ may be selected (Figure 2). A counterintuitive pulse scheme that makes use of the same two-pulse, two-color condition used in the previous examples would result in a potential control mechanism. STIRAP depends on the coherent adiabatic transfer between initial and final states in a two-color mechanism where the transient population of an intermediate state could be minimized. For example, by tuning a Stokes field to stimulated emission pumping, the STIRAP mechanism could in principle optimize a nonadiabatic transfer by reducing unproductive internal conversion that normally proceeds via the excited-state intermediate. Experimental methodology and theoretical methodology in this field are very challenging, and effective coherent control of photoisomerization via STIRAP is yet to be demonstrated. In practice, multilevel systems arising in real systems could prevent efficient STIRAP, and a detailed understanding of decoherence and detuning would be needed to advance this type of control. As with other coherent control mechanisms, evidence should be fully developed in the laser laboratory using ultrafast spectroscopy measurements prior to proposal and execution of a STIRAP-type TR-SFX experiment. It is included as a potential future application in particular with the anticipated increased sensitivity from high repetition rate XFEL instruments.

Another extension of the pump–probe ISRS equivalent using XFEL diffraction considers the application of multipulse trains, with a phase-stable continuous delay between pulses. This proposal follows the celebrated example from Keith Nelson who demonstrated the amplification of coherent nuclear motion using such shaped pulse trains.^101^ Timed sequences of repetitive femtosecond pulses have been used to “push” molecular vibrational modes and amplify the displacements as a result. This is proposed as a viable method in order to increase the effective displacement of modes that have an intrinsically small displacement. Such pulse trains could potentially allow the detection of vibrational modes that would have insufficient displacement to allow for the detection using single pulse excitation.

The examples described here and summarized in Figure 2 represent only a basic overview and introduction of nonlinear optical spectroscopy techniques that can be combined with ultrafast X-ray experiments. They introduce the possibility of a new field of structural dynamics research that can take advantage of the highly developed methodologies of ultrafast optical spectroscopy. Because of the multipulse synthesis and large amount of data that needs to be collected, which is typically much more than single pump–probe conditions, these applications are ideally tailored to the capabilities of high repetition rate XFEL instruments.

## 5 XFEL Beamline Optical Instrumentation for Structural Dynamics

**Figure 3:**
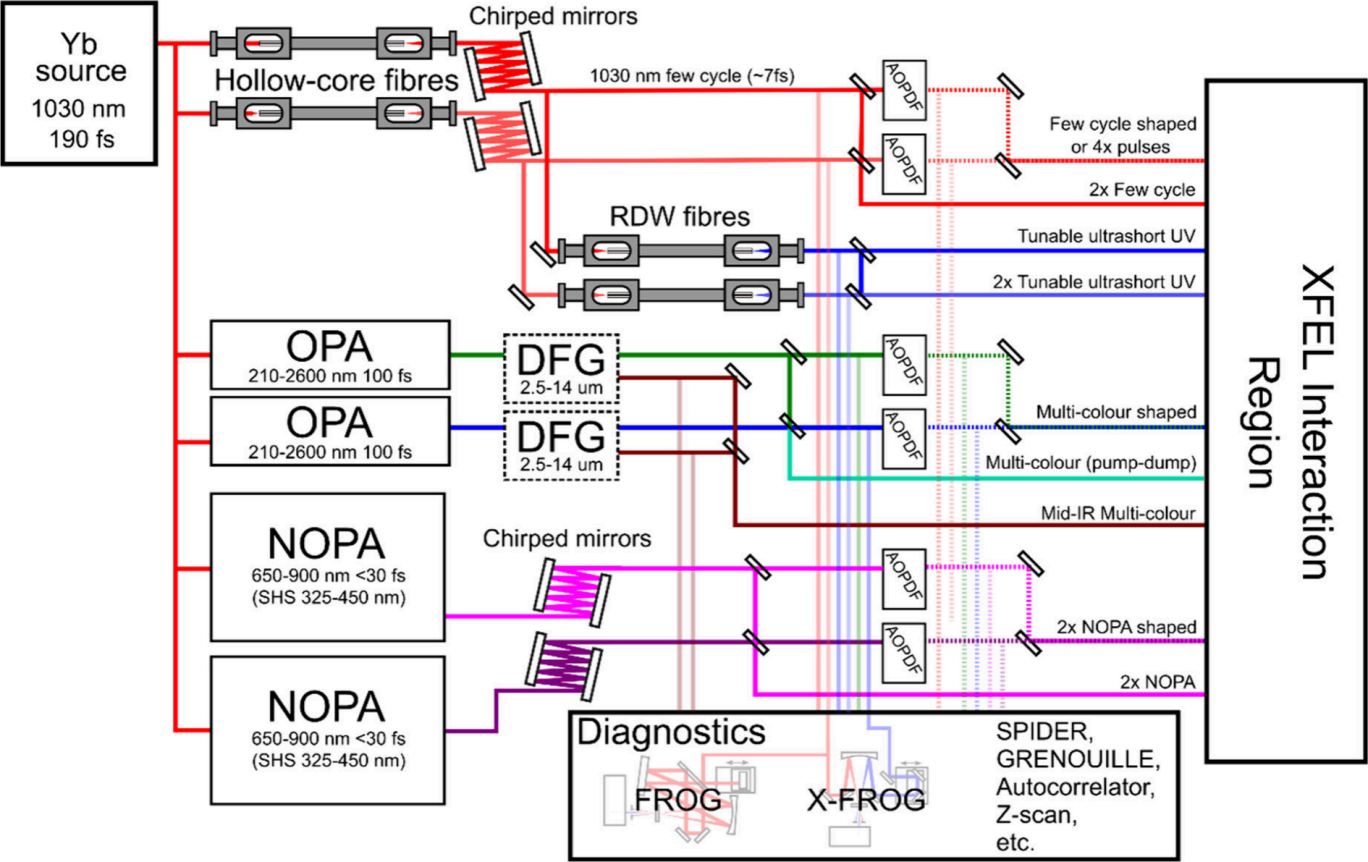
Proposed optical arrangement of systems and diagnostics available at an ideal ultrafast XFEL beamline, shown here with an example of an Yb pump laser. Operation at MHz repetiton rates would add further design considerations. The figure is representative of possible systems that could be present at a beamline, and it is not the expectation that a single fundamental pump would supply all systems pictured simultaneously but rather different combinations thereof, depending on the desired optical excitation scheme as detailed in Figure 2.

The following summarizes proposed light sources and control and characterization instrumentation that we believe should be standard for time-resolved XFEL beamlines in order to allow the execution of the various types of nonlinear spectroscopy experiments that we have discussed. We emphasize that there cannot be one single type of selected light source that will satisfy all of the conditions. Furthermore, frequency conversion methods have different limits of the maximum possible repetition rate. We argue that there should be maximal flexibility in the synthesis, shaping, stability, and characterization of all possible laser fields that can be utilized for time-resolved XFEL experiments (Figure 3). XFEL beamline users should be able to specify carrier frequency, power density, pulse duration, number of pulses, number of different carrier frequencies, interpulse phase stability, spectral and temporal phases, and spatial chirp characteristics. In order to consider the instrumentation that would be required, we propose to place a limitation to specify two different carrier frequencies. Experiments that would need more than two would be considered exotic and beyond standard beamline requirements, whereas many experiments would need two different carrier frequencies (Figure 2). In addition, we consider the pump laser characteristics, which determine the selection of the frequency conversion mechanisms. This selection will strongly depend on the repetition rate of the XFEL. For superconducting technology machines that will allow continuous wave MHz repetition rates such as LCLS-II, the pump laser will ideally need to be an OPCPA, although solutions based on the nonlinear compression of Yb lasers could provide some of the proposed capabilities. For low repetition rate machines such as SwissFEL, SACLA, PAL-XFEL, and LCLS-I, the pump laser of choice would be based on either titanium:sapphire or Yb technology.

The LCLS-II OPCPA “NIR-OPCPA”^102,103^ output (as detailed during the time of writing^104^) is a suitable source to consider the frequency conversion. NIR-OPCPA is ∼60 nm fwhm centered at 800 nm (∼740–870 nm at the few percent level) and is therefore comparable with the fundamental of titanium:sapphire lasers. At a 93 kHz repetition rate, the pulse energy reaches approximately 0.4 mJ (∼1 mJ at 33 kHz) with a duration of ∼17 fs, marking the transport limit on dielectric mirrors from the laser hall to the end stations. To use such a source for the highest possible flexibility, three types of light sources, and two of each, would satisfy most requirements:

i) Two commercial collinear OPAs with frequency mixing options (SFG, SHG, and FHG) for NIR, visible, and UV generation. These light sources would be optimized for conversion efficiency and deliver modest bandwidth and pulse durations. Such systems are ideal for experiments that require very specific pump wavelengths, which vary the pump wavelength during the beamtime or large pulse energy in order to maximize the concentration of transient populations. Difference frequency generation (DFG) stages can also be added to extend the range of wavelengths to the mid-IR (2.5–14 μm). DFG also has the added advantage of the pulses from DFG being CEP-stable.
ii) Two noncollinear OPAs (NOPAs) with SFG and SHG extensions in order to support larger bandwidth and short pulse operation in the visible region at the expense of pulse power. We suggest configuring one NOPA as single stage, typically providing up to 10 μJ pulse energy, and one two-stage NOPA to offer higher power but typically at the expense of some bandwidth. Pulse durations after compression will be on the order of 7–10 and 15–25 fs, respectively, and would allow propagation in air, liquid, and/or suspension sample environments and the use of transport and focusing optics as required. These sources are ideal for experiments that achieve high time resolution and for the synthesis of phase-locked pulse pairs (Figure 2).
iii) Hollow capillary fiber extensions to existing system can produce pulse durations down to a few femtoseconds^105,106^ and provide tunability from the deep UV to the near-IR. Such systems are not easily retrofitted onto existing beamlines, especially for a single experiment due to the setup, alignment, and fragility and hence low transportability of existing systems. Well-optimized and regularly used residential systems would be highly preferable as in additional few-cycle pulses, particularly those reaching into the deep UV, requiring in-vacuum integration and propagation as well as an in-vacuum target chamber. The large bandwidths and susceptibility to dispersion would also benefit greatly from full pulse characterization.
iv) Pulse shapers such as acousto-optic programmable dispersive filters (AOPDFs) allow control of the spectral phase of a pulse and the ability to manipulate its temporal structure. They are ideal tools for creating some of the optical pulse schemes which are required to perform some the experimental approaches listed in Figure 2. This includes creating pulse pairs with variable separation and adding spectral chirp to compress/stretch pulses or higher-order phases to create exotic pulse structures. AOPDFs can be rapidly switched, allowing shot-to-shot variation in pump conditions during data collection or simply pulse picking. The amplitude of the acoustic wave can also be adjusted to perform a power titration rapidly. As such, it would be possible to preprogram various experimental pump conditions and collect them all during the same run, similar to the way “light” and “dark” data are currently interleaved during TR-SFX experiments. This has several advantages over data collection of sequential runs as it can minimize the impact of systematic variations and slow drifts that can occur during an extended experimental data collection, all while making more efficient use of the new high data rates.
  AOPDFs do come with a pulse energy cost as only a portion of the incident pulse is diffracted by the acoustic wave, leading to ∼40–60% efficiency (wavelength- and bandwidth-dependent). Additionally, commercially available AOPDFs are currently limited to a few tens of μJ and ∼10 kHz. However, an AOPDF can be introduced and removed with simple flip mirrors, and its general implementation can be accomplished without modification of the upstream optics and primary laser system.
v) All excitation sources should be fully characterized both temporally and spatially as standard during normal beamtime operation. Femtosecond pulse characterization techniques such as frequency-resolved optical gating (FROG) and spectral phase interferometry for direct electric-field reconstruction (SPIDER) as well as the more advanced techniques derived therefrom recover both the spectral and temporal phases of the optical pulses. This is not the case for more rudimentary characterization techniques such as autocorrelation and cross correlation which provide only the pulse envelope. More advanced FROG variants such as the GRENOUILLE^107^ are single-shot devices which can provide information about the shot-to-shot phase, a parameter that is not normally measured but could be critical for measurements which depend on the carrier-envelope phase (CEP) or experimental schemes using a pulse shaper described above. Since the detector for a GRENOUILLE setup is simply a 2D optical camera, its repetition rate is limited only by the frame rate of the camera which can be binned depending on the desired resolution of the measurement at the trade off with frame rate. Such measurements could become the standard for XFEL beamlines where the femtosecond optical pump-pulse structure is measured for every laser shot in a similar way to the X-ray photon spectrum. The pulse energy and synchronization timing tool signals are currently routinely measured for every X-ray shot at the XFEL facilities. We note that one downside of a GRENOUILLE is that it requires a different nonlinear crystal for each significant change in the central pulse wavelength and as such will require different arrangements if using tunable wavelength outputs such as OPAs. Additionally, commercial cross-correlation devices exist that also allow for feedback loop compression using AOPDF shaping technology.
  It is also important not to neglect the spatial profile of the laser beam and focus. The significant difference between optical and X-ray focal spot sizes, typically ∼100 μm vs a few μm, results in the sample probed by the XFEL being illuminated only by a small subsection of the optical focus. As such, any high spatial frequency structure or chromatic aberration in the optical focus could lead to an unintended variation in pump conditions. Beam profile and focal spot measurements are simply implemented but should not be neglected.
  The timing between the optical pulse and the XFEL is of significant consideration for ultrafast measurements. XFELs are prone to jitter between the optical and X-ray pulses with distribution widths typically a few hundred fs (at LCLS) but reported as smaller than 25 fs at PAL-XFEL. This timing jitter is addressed through the use of timing tools which use spatial^108^ or spectral^109^ encoding to measure the jitter shot to shot and then postprocess data sorting. Improvements in synchronization have been seen in recent years at SACLA where their new synchronization system reduced arrival jitter to <50 fs,^58^ removing the need for timing tools for data sorting for all but the highest temporal resolutions. However, with the use of very short few-cycle pulses, higher resolution timing tools may still be necessary for techniques such as chirped Laue,^110^ which can potentially push beyond the temporal limit of the X-ray pulse envelope and move toward few femtosecond or even attosecond dynamics. Techniques other than transient reflectivity should be investigated in order to develop better cross-correlation accuracy for arrival time determination.
vi) Z-scan measurements are an additional characterization technique that is highly recommended. Such systems have a very simple design and implementation and will allow beamline users to verify the essential optical parameters for their samples. In addition to pulse characterization, the Z-scan technique will allow users to copy and reproduce the home laboratory conditions as close as possible at the experimental end station. Such measurements involve scanning the target through a laser focus with constant flux to measure transmission of the sample at fixed pulse energy but increasing intensity. When employed at or near saturation, it can resolve nonlinear excited state absorption and the saturable absorption of a sample, which are critical parameters to achieving suitable excitation conditions and ensuring the success of a TR-SFX experiment which requires photoexcited populations of the target state >10% to be reliably resolved.

## 6 Conclusions

We have summarized possible future classes of advanced experiments that could be employed at XFEL beamlines in order to measure the nonlinear molecular response that involves electronic and vibrational coherences from optical excitation. Such experiments take advantage of the fact that the required optical laser pulses can be set up separate from XFEL beamline instrumentation that typically uses single X-ray pulses for time-resolved measurements. The proposed experiments utilize a more complex illumination scheme than those currently available at operation facilities at the time of writing. They take full advantage of an increased repetition rate and subsequent data rates to reveal phenomena not previously accessible by conventional pumping. We detail the recommendations for the possible laser system and light source arrangements that could supply these pulse schemes as well as properly characterize pump pulses and samples for these measurements.
